# The Predictive Role of C-Reactive Protein, Leukocyte Cell Count, and Soluble Urokinase Plasminogen Activator Receptor for Pulmonary Sequelae in Hospitalized COVID-19 Survivors: A Prospective Single-Center Cohort Study

**DOI:** 10.3390/jcm14051717

**Published:** 2025-03-04

**Authors:** Izzet Altintas, Thomas Kallemose, Mette Bendtz Lindstrøm, Imran Parvaiz, Iben Rokkedal, Lene Juel Rasmussen, Katrine Kjær Iversen, Jesper Eugen-Olsen, Kasper Karmark Iversen, Ejvind Frausing Hansen, Charlotte Suppli Ulrik, Jan Olof Nehlin, Ove Andersen

**Affiliations:** 1 Department of Clinical Research, Copenhagen University Hospital Amager and Hvidovre, 2650 Hvidovre, Denmark; thomas.kallemose@regionh.dk (T.K.); mette.bendtz.lindstroem@regionh.dk (M.B.L.); jespereugenolsen@gmail.com (J.E.-O.); jan.nehlin@regionh.dk (J.O.N.); ove.andersen@regionh.dk (O.A.); 2 Emergency Department, Copenhagen University Hospital Amager and Hvidovre, 2650 Hvidovre, Denmark; imran.parvaiz@regionh.dk (I.P.); iben.rokkedal.02@regionh.dk (I.R.); 3 Center for Healthy Aging, Department of Cellular and Molecular Medicine, University of Copenhagen, 2200 Copenhagen, Denmark; lenera@sund.ku.dk; 4 Department of Infectious Diseases, Copenhagen University Hospital Amager and Hvidovre, 2650 Hvidovre, Denmark; katrine.kjaer.iversen@regionh.dk; 5 Emergency Department, Copenhagen University Hospital Herlev and Gentofte, 2730 Herlev, Denmark; kasper.karmark.iversen@regionh.dk; 6 Department of Clinical Medicine, University of Copenhagen, 2100 Copenhagen, Denmark; charlotte.suppli.ulrik@regionh.dk; 7 Department of Respiratory Medicine, Copenhagen University Hospital Amager and Hvidovre, 2650 Hvidovre, Denmark; ejvind.frausing.hansen@regionh.dk

**Keywords:** SARS-CoV-2, COVID-19, pulmonary function impairment, DL_CO_, prediction, CRP, leukocyte cell count, suPAR

## Abstract

**Highlights:**

**What is already known on this topic?**

- There is an urgent clinical need for biomarkers to predict pulmonary deterioration among acutely admitted patients with COVID-19. Patients with COVID-19 present with a wide range of clinical outcomes, from mild illness to respiratory failure. Identifying patients who are at risk of deterioration early on can guide clinical management, resource allocation, and targeted interventions.

**What does this study add?**

- This study shows that the most commonly affected pulmonary function parameter during follow-up was DLCO impairment. Among the biomarkers studied, soluble urokinase Plasminogen Activator Receptor (suPAR) at admittance demonstrated the strongest correlation with DLCO impairment, and a low suPAR cut-off value showed the highest negative predictive value (NPV) for DLCO impairment.

**How might this study affect research, practice, or policy?**

- This study could assist physicians in reducing the number of patients requiring follow-up at pulmonary outpatient clinics, particularly due to the high negative predictive value (NPV) of the biomarkers in forecasting DLCO impairment. This could potentially be of benefit for individual patients and, at the same time, alleviate the pressure on the healthcare system.

**Abstract:**

**Background:** Pulmonary function impairment significantly affects quality of life, work ability, and healthcare utilization. Among patients with COVID-19, respiratory symptoms vary in severity. This study aimed to assess whether biomarkers related to respiratory function and inflammation at emergency department (ED) admittance can predict long-term pulmonary function impairment in COVID-19 survivors. **Methods:** This prospective single-center study recruited patients 4–5 months post-COVID-19 infection using consecutive sampling. All attendees at the respiratory outpatient clinic were invited to participate. Pulmonary function tests, including diffusing capacity of the lungs for carbon monoxide (DL_CO_), total lung capacity (TLC), forced expiratory volume in the first second (FEV1), and forced vital capacity (FVC), were performed, with DL_CO_ < 80% as the key indicator of impairment. Baseline biomarkers—C-Reactive Protein (CRP), leukocyte counts, and soluble urokinase Plasminogen Activator Receptor (suPAR)—were correlated with post-discharge DL_CO_ values. **Results:** This study enrolled 110 patients with COVID-19; 58.2% were female, the median age was 61.5, and the average BMI was 27.2. Smoking history showed that 53.7% were never smokers, 43.5% were former smokers, and 2.8% were current smokers. A diffusion deficit (DL_CO_ < 80%) was present in 48.6% of patients. Leukocyte counts and suPAR had the highest sensitivity (>0.80) for predicting DL_CO_ impairment but showed low specificity and a positive predictive value (PPV) of around 0.50. However, combining all biomarkers improved prediction accuracy, with a negative predictive value (NPV) of 0.93. **Conclusions:** The chosen inflammatory biomarkers by themselves had a limited ability to predict long-term pulmonary function impairment in COVID-19 survivors. However, when combined, they demonstrated a high negative predictive value (NPV) for identifying DL_CO_ impairment. This strategy could help clinicians better tailor follow-up care for patients with COVID-19.

## 1. Background

Some COVID-19 survivors may experience symptoms following infection with severe acute respiratory syndrome coronavirus 2 (SARS-CoV-2). Long COVID, which follows the post-acute sequelae of SARS-CoV-2 infection (PASC) [1], refers to a condition where individuals continue to experience symptoms or develop new ones well beyond the acute phase of COVID-19 illness. In March 2023, an article discussed the long-term sequelae following COVID-19 infection, impacting at least 65 million individuals worldwide [2]. The most frequently reported persistent symptoms among patients with long COVID-19 include fatigue, dyspnea, cough, memory loss, chest or muscle pain, phlegm, attention loss, and a loss of smell/taste. More than 651 million cases of COVID-19 have been documented, with the actual number likely to be even higher due to many undocumented cases [2]. In an international long COVID cohort study, the overall symptom complex involved 10 organ systems, encompassing an estimated total of 203 different symptoms [3]. Pulmonary function impairment, assessed by the lower diffusing capacity of the lungs for carbon monoxide (DL_CO_), forced expiratory volume in the first second (FEV_1_), forced vital capacity (FVC), and total lung capacity (TLC), among patients with COVID-19 has already been reported [4,5,6,7].

Patients with COVID-19 can exhibit different clinical manifestations, such as excessive clotting, inflammation, and endothelial cell damage. The clinical manifestations of COVID-19 can present beyond respiratory symptoms and affect multiple organ systems. These are known to include hypertension, diabetes, and ischemic heart disease and are linked to an increased risk of mortality. Additionally, patients with COVID-19 frequently experience a broad spectrum of arrhythmias, heart failure, and thrombotic events such as venous thromboembolism [8]. These features may lead to respiratory failure in certain cases [9]. The lungs are the primary target of COVID-19, with damage primarily affecting alveolar epithelial and endothelial cells, hindering tissue repair and fibrinolysis and causing cellular senescence. This results in secondary fibroproliferation [7,10] hyaline membrane formation, capillary damage, bleeding, fibrous proliferation of alveolar septa, and pulmonary consolidation, as documented in previous studies [11]. Abnormalities in respiratory function, computed tomography (CT) [12,13], or magnetic resonance imaging [14] were found among survivors of COVID-19 pneumonia months after hospitalization.

A previous study from 2021 showed that the chronic inflammation biomarker soluble urokinase Plasminogen Activator Receptor (suPAR) serves as a robust indicator for identifying patients with COVID-19 who will not develop respiratory failure and, therefore, may not require treatment with mechanical ventilation [15]. In patients experiencing the post-acute sequelae of SARS-CoV-2 (PASC), or long COVID following the PASC, it was shown that immune system cells infiltrating lung tissue were associated with increased systemic inflammation and reduced lung function [16,17], and the overexpression of inflammatory biomarkers, including CRP and the neutrophil-to-lymphocyte ratio (NLR), was associated with subsequent lung function abnormalities in patients who recovered from COVID-19 [18].

The primary aim of this study was to investigate whether measures of blood biomarkers in patients with COVID-19 upon admittance to the ED could predict long-term pulmonary function impairment among COVID-19 survivors. We included the acute inflammation marker C-Reactive Protein (CRP), leukocyte cell count, and the chronic inflammation biomarker suPAR to explore their predictive value for impaired DL_CO_ among patients with COVID-19 4–5 months post-discharge.

## 2. Methods

### 2.1. Study Design and Setting

This study is based on data from a prospective longitudinal cohort study involving patients who recovered from COVID-19 after hospitalization [3]. The study included a heterogeneous group of patients ranging from needing treatment with oxygen supplementation by a nasal cannula, continuous positive airway pressure (CPAP), and invasive mechanical ventilation. The study inclusion period was 1 March to 1 November 2020. The study was conducted at the 685-bed Copenhagen University Hospital, Amager and Hvidovre, Denmark, with a catchment area of approximately 550,000 citizens. Access to electronic health records (EHRs) was granted by the hospital data protection manager.

### 2.2. Patients

**Figure 1:**
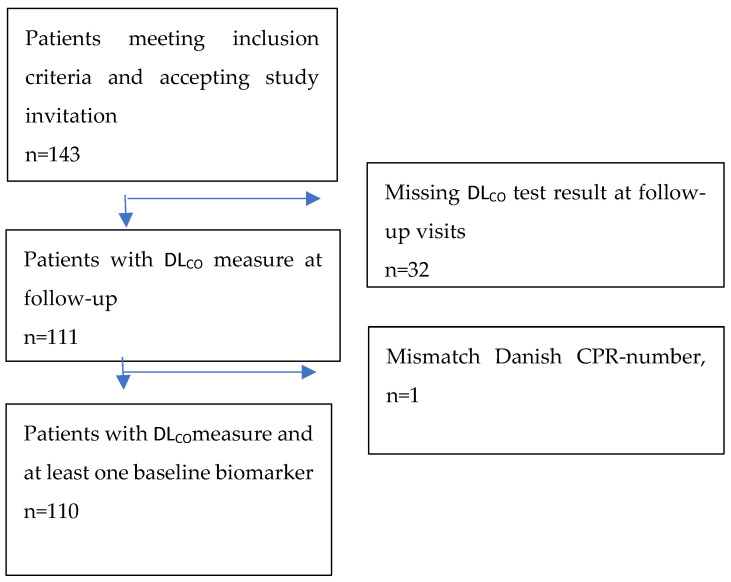
Diagram illustrating the enrollment of patients in the study and their inclusion in the analysis. Abbreviations: DL_CO_: diffusing capacity of the lungs for carbon monoxide, CPR: central person register.

Patients were recruited upon attending their follow-up visits at the Department of Respiratory Medicine 4–5 months after experiencing COVID-19 infection. Patients were eligible for inclusion if they fulfilled the inclusion criteria: (1) hospitalized at the Department of Respiratory Medicine or Department of Infectious Diseases with COVID-19 between March 1st and November 1st, 2020; (2) SARS-CoV-2 infection verified by Reverse Transcription Polymerase Chain Reaction (PCR); (3) primary reason for being hospitalized was a diagnosis of COVID-19; and (4) evidence of COVID-19 pneumonitis on chest X-ray or computed tomography scan and/or requiring supplemental oxygen by nasal cannula, continuous positive airway pressure (CPAP), or invasive mechanical ventilation. The exclusion criteria were (1) age < 18 years, (2) not able to attend the follow-up visit due to severe disability or chronic cognitive deficits, and (3) deceased prior to the intended time of follow-up. The study’s patient flow is illustrated in Figure 1. Our study follow-up program aligned with standard rehabilitation visits at the Department of Respiratory Medicine.

All patients in this study were naïve to COVID-19 vaccination, as Denmark’s vaccination program began only after the European Medicines Agency (EMA) granted approval on 21 December 2020.

### 2.3. Variables and Measurements

The post-discharge diffusing capacity of the lungs for carbon monoxide (DL_CO_) was chosen as the primary clinical endpoint. DL_CO_ measures the ability of the lungs to transfer gases from the air into the bloodstream and is primarily used to evaluate gas exchange in conditions such as interstitial lung disease, emphysema, and pulmonary vascular diseases [19].

We assessed DL_CO_ during follow-up by using the single-breath method [19]. Spirometry with measurements of the forced vital capacity (FVC) and the forced expiratory volume in the first second (FEV_1_) was performed according to the standard procedure [20]. Additionally, DL_CO_ was defined as impaired if DL_CO_ was <80% of the predicted value or as non-impaired. All measurements were performed using a digital spirometer, EasyOnePro (Medizintechnik AG, Zürich, Switzerland) [21,22].

Predictive variables included biomarkers of both acute and chronic inflammation: CRP, leukocyte cell counts, and suPAR were measured at the time of initial hospitalization for COVID-19. A standard panel of admission blood samples was analyzed at the Department of Clinical Biochemistry, Copenhagen University Hospital, Hvidovre. CRP was measured using a COBAS 8000 analyzer (Roche Diagnostics, Mannheim, Germany). Leukocyte cell counts were measured using flow cytometry on a Sysmex XN 9000 (Sysmex Corporation, Kobe, Japan). Plasma suPAR was measured using an suPARnostic^®^ AUTO Flex ELISA (ViroGates, Birkerød, Denmark) by experienced technicians blinded to the clinical data. The suPARnostic assay has a low detection limit of 100 pg/mL and intra- and inter-assay variations of 2.75% and 9.17%, respectively, as determined by the assay manufacturer.

CRP, leukocyte cell counts, and suPAR are reported as continuous measurements and were also categorized into three intervals (low, middle, and high). CRP intervals were defined as <50 (mg/L) (low), 50-100 (mg/L) (middle), and > 100 (mg/L) (high), and leukocyte cell count intervals were defined as <3.5 10^9^/L (low), 3.5 10^9^/L–8.8 10^9^/L (middle), and >8.8 × 10^9^/L (high). These reference intervals are routinely used at the Department of Clinical Biochemistry, Copenhagen University Hospital, Hvidovre, Denmark. suPAR intervals were categorized as <4 ng/mL (low), 4–6 ng/mL (middle), and >6 ng/mL (high), as these levels have already been investigated in a prior study in patients with COVID-19 [15]. Elevated suPAR levels have previously been demonstrated to predict adverse clinical outcomes, including the need for mechanical ventilation [23]. Slightly more than half of the cohort had a suPAR measurement performed.

The demographics, clinical data, and comorbidities of the included patients were obtained from the electronic health records at index hospitalization.

### 2.4. Patients and Public Involvement

Patients and/or the public were not involved in the design, conduct, reporting, or dissemination plans of this research.

### 2.5. Patient Consent for Publication

As this was a standard of care non-interventional study, formal approval from the ethics committee was not required. However, the Ethics Committee in the Capital Region of Denmark was informed about the study (jr.nr. H-20040749). No informed consent was required.

### 2.6. Statistics

Demographic data are presented using proportions for categorical values, and continuous variables are presented as the mean with standard deviation (SD) or median values with interquartile ranges (IQRs) depending on the distribution. Pulmonary function parameters are presented as mean values with standard deviations.

The correlation between biomarker levels and DL_CO_ percentage was estimated using Pearson correlation with 95% confidence intervals (CIs) and tested against a null hypothesis (H0) value of 0.

The distribution of biomarker interval categorization (low, middle, and high) and DL_CO_ < or > 80% is presented in terms of proportions. The sensitivity, specificity, positive predictive value (PPV), and negative predictive value (NPV) of DL_CO_ < 80% according to biomarker cut-offs were estimated. Cut-offs were defined for the middle and high categories compared to the low (referred to as a ‘low cut-off’) and for the high categories compared to the middle and low (referred to as a ‘high cut-off’). The 95% CIs were estimated by exact binomial confidence intervals. To assess the combined predictive value of the biomarkers, we performed a receiver operating characteristic (ROC) analysis that included all three biomarkers. We used the probability of pulmonary functional impairment, estimated from a logistic regression model that incorporated continuous biomarker measurements, as a threshold scale in our analysis. Areas under the curve (AUCs) for the ROC analysis are presented with 95% CIs and *p*-values for tests with a null hypothesis AUC of 0.5.

A sensitivity analysis excluding all patients with pre-existing asthma or chronic obstructive pulmonary disease (COPD) was also performed. As the cut-off for the combined analysis was data-dependent, the analysis was performed for both the new cut-off and the cut-off from the original analysis.

Variables were included with all available measures in their respective analysis; no further handling of missing data was performed.

The statistical program version 4.1.0 (R Foundation for Statistical Computing, Vienna, Austria) was used for analyses and figures. A *p*-value below 0.05 was considered significant. The exact binomial confidence intervals were estimated using the epiR package.

## 3. Results

We analyzed data from a prospective longitudinal cohort study involving patients who recovered from COVID-19 after hospitalization. Of the 160 invited patients with COVID-19, a total of 143 patients agreed to participate in the present standard of care observational cohort study, and of these, 111 had DL_CO_ measured during follow-up. Below, Figure 1 depicts a summary of patient enrolment in this study.

The baseline clinical characteristics and demographic data obtained at hospital admission among patients with COVID-19 are shown in Table 1. The subgrouping of participants based on DLCO < 80% vs. ≥80% and further classification by age < 60 vs. >60 years is also presented in Table 1. Out of the 110 patients, 64 (58.2%) were female, and the median age was 61.5. There were no previous hospitalizations due to severe respiratory or cardiovascular diseases among the enrolled patients. Twelve patients had heart failure, ten patients had asthma, and seven had COPD. The median packyear of tobacco use was 18, with 53.7% classified as never, 43.5% as former, and 2.8% as current tobacco users. The median BMI was 27.2 kg/m^2^ (Table 1). In total, 8.6% of the patients were treated with continuous positive airway pressure (CPAP), and 3.9% received invasive mechanical ventilation (not shown in the table). The overall median length of stay was 6 days (IQR: 3–10 days). The most frequent comorbidities are listed in Table 1.

The pulmonary functional parameters, such as forced expiratory volume in 1 s (FEV_1_), forced vital capacity (FVC), DL_CO_, and total lung capacity (TLC), were assessed at the respiratory outpatient clinic 4–5 months post-discharge, and they are presented in Table 2. The mean and standard deviations for the percent predicted variables FEV_1_, FVC, DL_CO_, and TLC are provided. A reduction of less than 80% was observed for FEV_1_ in 23.9% of patients (*n* = 26), for FVC in 15.6% of patients (*n* = 17), for TLC in 28.2% of patients (*n* = 31), and for DL_CO_ in 45.5% of patients (*n* = 50) (Table 2). The average FEV_1_/FVC ratios were 0.76 (SD = 0.16) for patients with DL_CO_ < 80% and 0.79 (SD = 0.06) for patients with DL_CO_ > 80%.

### Biomarker Correlation with DL_CO_

The correlation coefficients for all biomarkers were not statistically significant. CRP and leukocyte cell count showed values of 0.04 (CI: −0.14 to 0.23) and 0.05 (CI: −0.14 to 0.24), respectively, with all *p*-values ≥ 0.05.

For all biomarkers, the sensitivity ranged from 0.52 to 0.88 at low cut-offs, with specificities ranging from 0.07 to 0.45. While the sensitivity did not exceed 0.31, the upper cut-off specificity ranged from 0.76 to 0.80 (Table 3). All cut-offs had similar PPVs, with none exceeding 0.56. In terms of the NPVs, leukocyte cell counts at the low cut-off showed the lowest value at 0.40, while suPAR at the low cut-off exhibited the highest value at 0.72. All other cut-off points showed values around 0.50 (Table 3). It is worth noting that the CI for all these estimates was relatively wide. The combination of biomarkers notably improved the NPV, with the most significant enhancement reaching a value of 0.93, while the other measures showed relatively smaller improvements (Table 3). The AUC for the ROC analysis of the combination of biomarkers was 0.71 (CI: 0.58–0.83, *p* = 0.004).

The sensitivity analysis showed similar results, with the only difference found for the combined analysis with thresholds based on data for patients with no pre-existing asthma or COPD, showing reduced sensitivity and NPVs (Supplementary Table S1).

When evaluating the biomarkers in patients with DL_CO_ < 80%, most CRP measurements fell within the lower category (<50 mg/L), whereas the suPAR measurements (4–6 ng/mL) and leukocyte cell counts (3.5–8.8 × 10^9^/L) exhibited the highest proportion of patients within the intermediate category. For patients with DL_CO_ ≥ 80%, the CRP measurements were still predominantly within the low category (≥50 mg/L), the leukocyte counts revealed the largest proportion of patients within the intermediate category (3.5–8.8 × 10^9^/L), and the suPAR measurements were equally distributed between the low (<4 ng/mL) and intermediate categories (4–6 ng/mL) (Table 4).

## 4. Discussion

Among patients with COVID-19 who attended follow-up visits at the outpatient clinic and agreed to participate in this study, DLCO impairment was the most commonly affected pulmonary function parameter observed over time. Among the biomarkers analyzed, suPAR demonstrated the strongest correlation with DLCO measurements, and a low suPAR cut-off value yielded the highest negative predictive value for DLCO impairment.

The FEV_1_/FVC ratio is typically used to assess airflow obstruction in the lungs: a normal FEV_1_/FVC ratio is usually greater than 0.70. A ratio of less than 0.70 is often used to define airflow limitation. In this study, the ratio was on average higher than 0.70 for both DL_CO_ < 80% and >80%. When a reduced DLCO is caused by chronic airflow limitation, such as in emphysema, it will be associated with a low FEV1/FVC, whereas a low DLCO caused by interstitial lung abnormalities will be associated with a normal or high FEV1/FVC ratio. This, combined with the low prevalence of COPD in the cohort, indicates that the likely cause of DLCO impairment was interstitial abnormalities due to COVID-19.

Of the 110 patients with COVID-19 who were evaluated, no correlation was found between baseline measured biomarkers and impaired pulmonary functional capacity assessed by DL_CO_ measurement 4–5 months post-discharge at the respiratory outpatient clinic. When predicting DL_CO_ impairment using biomarker cut-off values, the low cut-off exhibited the highest sensitivity but low specificity, while the high cut-off had low sensitivity and high specificity. This could be attributed to the large number of patients with biomarker values falling within the middle category, which represents the difference between high and low cut-offs. The DLCO impairment/non-impairment distribution is also quite similar in the middle groups. An additional subdivision of the middle group could result in a clearer separation of DLCO impairment/non-impairment, which could result in better performance. However, as the current grouping is based on pre-existing cutoffs with none available for further subdivision, any subdivision would be somewhat arbitrary. The performance values suggest that the cut-off values are effective at identifying either the most impaired individuals (low cut-off) or the most non-impaired individuals (high cut-off) but are less effective at identifying patients in the middle group. Both PPVs and NPVs for most cut-offs hovered around 50%, suggesting that at least half of these allocations were misclassifications.

The integration of all included biomarkers yielded the most substantial improvement, resulting in an NPV of 0.93. This suggests that nearly all non-impaired patients were accurately classified. This could indeed assist physicians in reducing the number of patients requiring follow-up in the clinical setting. However, the specificity remained low at 0.41, indicating that less than half of all non-impaired patients were correctly identified.

A follow-up study by Fortini et al., 2021, investigated symptoms and pulmonary alterations in COVID-19 survivors (*n* = 105) from 3 to 6 months post-discharge [24]. This study revealed persisting diffusing capacity alterations in the lungs, which is consistent with our findings. The DL_CO_ impairment compromised gas diffusion between the alveoli and pulmonary capillaries, possibly due to epithelial and endothelial pulmonal damage or a damaged intra-alveolar diffusion pathway. In clinical practice, reduced DL_CO_ values are often observed in conditions involving extensive interstitial fibrosis or vascular obstruction. Low DL_CO_ levels may indicate various conditions, including diffuse parenchymal pulmonal disease, reduced vasculature and alveolar tissue (as seen in chronic obstructive pulmonary disease, COPD), or pulmonary vascular diseases (such as pulmonary embolism, chronic thromboembolic pulmonary hypertension, or idiopathic pulmonary arterial hypertension) [25,26]. Histologic findings in the lungs have shown alveolar damage with hyaline membrane formation, alongside microthrombi in small pulmonary vessels [27]. We therefore hypothesize that patients with COVID-19 could experience a course characterized by a combination of interstitial pneumonia with edema and micro/macro-thrombosis [28]. This finding further supports the notion that DL_CO_ is a particularly sensitive tool for monitoring patients with COVID-19 during follow-up.

The association between the biomarker suPAR and asthma, as well as suPAR and COPD patients, regarding the readmission rate and mortality, had already been described before the COVID-19 pandemic [29,30]. Asthma is not normally associated with a reduction in DL_CO_, and the low prevalence of COPD (6.4%) in the present study makes it less plausible that the decrease in pulmonary function parameters, especially DL_CO_, is due to chronic pulmonary conditions, such as COPD. Therefore, COVID-19 could indeed be responsible for the clinical impairments observed in the study.

A systematic review and meta-analysis published by Kin Israel Novarte (2022) and colleagues aimed to identify the risk factors that could predict the development of long COVID. Among 1978 studies, the authors included 37 peer-reviewed studies and 1 preprint. Old age appeared to be associated with long COVID symptoms; however, the meta-analysis did not reveal a significant association between old age and long COVID. The study concluded that female sex was associated with long COVID symptoms, and this was confirmed by the meta-analysis. Additionally, medical comorbidities such as pulmonary disease, diabetes, obesity, and organ transplantation were identified as potential risk factors for long COVID [31].

A double-blinded, randomized controlled phase 3 trial, involving patients with COVID-19 with suPAR levels above 6 ng/mL, who were randomized to receive anakinra (IL-1 receptor antagonist) treatment or a placebo, showed protection against COVID-19 progression among the patients treated with anakinra. Whether anakinra also mitigates the long-term decline in DL_CO_ and other pulmonary function parameters is currently unknown [32].

In the early phase of the first wave of the COVID-19 pandemic, the clinical outcomes such as the development of respiratory failure and the need for mechanical ventilation among patients with COVID-19 with varying suPAR cut-off values were addressed [15,23]. Our previous studies further explored whether patients with COVID-19 developed respiratory failure requiring treatment with mechanical ventilation [13] and examined different disease trajectories [23]. Since long COVID presents a relatively new group of health problems, research is needed to elucidate this area and provide physicians with a better understanding of the molecular pathophysiology. Therefore, in the current study, we investigated three independent biomarkers, CRP, leucocyte cell count, and suPAR, to determine whether they could predict long-term pulmonary function impairment as part of long COVID-19 among survivors. Such knowledge can enable physicians to predict long-term impairment in patients’ health and can aid in designing a more comprehensive follow-up course, including potential treatment regimens [6,7,8,9]. Early intervention could be expected to help patients in managing their impairments and sequelae. However, whether the treatment will lead to a complete curative outcome remains to be elucidated.

Our study has both strengths and limitations that need to be addressed. In terms of strengths, the study is prospective, including patients across a range of severity levels and a comprehensive pulmonary function assessment. The lack of pre-hospitalization information regarding pulmonary function should also be mentioned. The absence of DL_CO_ measurements for patients who died before the follow-up visit introduced an immortal time bias into the study population. All predictive estimates relied on patients surviving until the follow-up time, a factor that cannot be determined at baseline. Additionally, it is important to mention that suPAR measurements were not part of the standard laboratory work at the ED on initial admission. Due to the high workload during the COVID-19 pandemic, suPAR measurements were unfortunately only conducted for the patients we were able to reach out to.

It can be speculated that the measured biomarkers from the admission index may be influenced by differences in illness severity within the study population, particularly in terms of who would develop pulmonary functional impairment and experience impaired DL_CO_ values at follow-up. Another study analyzed DL_CO_ (% predicted) at follow-up and found no correlation with CRP levels measured upon admission [33]. Further, we suggest that these biomarkers should be investigated in populations infected by later SARS-CoV-2 variants, as our findings may or may not apply to later variants or in populations vaccinated against COVID-19.

Since there were no baseline pulmonary function tests conducted among the hospitalized patients in this study, a pre-existing DL_CO_ impairment might have already been present in some of the patients with COVID-19. Without baseline DL_CO_ measurements before the onset of COVID-19, it is challenging to directly attribute the reduction in DL_CO_ solely to COVID-19. Several factors could contribute to decreased DL_CO_ in patients presenting with COVID-19, including pre-existing lung conditions, smoking history, age-related changes in lung function, and other concurrent illnesses or medications. However, despite the lack of pre-COVID-19 DL_CO_ measurements, certain aspects of the clinical presentation and disease progression suggest a potential association between COVID-19 and reduced DL_CO_. These aspects include the temporal relationship, the absence of other known causes, and consistency with the existing literature on the association between COVID-19 and decreased DL_CO_.

The results of this study have not been evaluated in an external sample to verify whether similar performance is reproducible or only applicable to the study sample.

## 5. Conclusions

This study found a low predictive value of baseline biomarkers for pulmonary sequelae in survivors of COVID-19 hospitalization. Biomarker cut-off values showed varying sensitivity and specificity, with the integration of all biomarkers improving the NPV but maintaining low specificity. Even so, this combined approach could assist physicians in reducing the number of patients requiring follow-up in the clinical setting, particularly given the high NPV attained by the combined biomarkers in predicting DLCO impairment.

## 6. Future Perspectives

Some patients with COVID-19 upon hospital discharge may develop pulmonary function impairment as part of long COVID 3 months post-discharge. Improving our understanding of the molecular mechanisms involved in disease progression and recovery, especially regarding lung function during convalescence, will contribute to identifying associated biomarkers for improved predictions of pulmonary impairment. Ongoing research is dedicated to unraveling these aspects.

The incidence of deep venous thrombosis (DVT) and pulmonary embolism among COVID-19 patients appears to be high [27,28]. These thrombotic events can lead to pulmonary vascular complications, such as pulmonary hypertension or vascular obstruction, which can impair gas exchange and affect DL_CO_ levels and other pulmonary function tests. In a single-center, prospective cohort study, we are currently recruiting patients with first-time lower extremity DVT consecutively at the emergency department of Copenhagen University Hospital, Hvidovre. Among the enrolled patients, we are measuring inflammatory, anti-inflammatory, and senescent cells and cells from the immune system to predict long-term complications, such as cardiovascular and cardiopulmonary complications in relation to the SARS-CoV-2 variant infection. The study has been registered at clinicaltrials.gov (NCT05789108).

## Figures and Tables

**Table 1:** Baseline description of patients with COVID-19 upon admission to the ED, *n* = 110.

Emergency Department: Variable (Median, IQR)	Level	Total (*n* = 110)	DL_CO_ < 80% (*n* = 50)	DL_CO_ > 80% (*n* = 60)	Age < 60 Years (*n* = 51)	Age > 60 Years (*n* = 59)
Age (years)		61.5 (50.0:73.0)	65.0 (54.2:75.0)	56.5 (48.8:68.2)	49.0 (43.5:54.0)	72.0 (65.0:79.0)
BMI		27.2 (23.7:30.5)	25.0 (23.0:29.6)	27.8 (24.9:31.0)	27.7 (24.8:30.8)	26.2 (23.3:29.6)
Sex	Female	64 (58.2%)	24 (48%)	40 (66.7%)	32 (62.7%)	32 (54.2%)
CRP (mg/mL) (*n* = 72)		55.0 (28.2:100.0)	55.0 (27.0:100.0)	55.0 (33.5:97.0)	55.0 (27.5:105.0)	55.0 (30.5:93.5)
Leukocyte Cell Count (*n* = 72)		6.4 (4.9:8.4)	6.4 (5.2:8.4)	6.3 (4.9:8.1)	6.4 (5.2:9.3)	6.2 (4.4:7.8)
suPAR (ng/mL) (*n* = 66)		5.1 (3.7:6.1)	5.4 (4.4:6.7)	4.6 (3.5:6.0)	5.0 (3.6:6.0)	5.1 (4.2:6.4)
Hypertension (*n*)		48 (43.6%)	21 (42%)	27 (45%)	15 (29.4%)	33 (55.9%)
Diabetes (*n*)		26 (23.6%)	12 (24%)	14 (23.3%)	13 (25.5%)	13 (22%)
Congestive Heart Failure (*n*)		12 (10.9%)	6 (12%)	6 (10%)	1 (2%)	11 (18.6%)
Atrial Fibrillation (*n*)		7 (6.4%)	4 (8%)	3 (5%)	0 (0%)	7 (11.9%)
Chronic Obstructive Pulmonary Disease (*n*)		7 (6.4%)	5 (10%)	2 (3.3%)	1 (2%)	6 (10.2%)
Asthma (*n*)		10 (9.1%)	5 (10%)	5 (8.3%)	3 (5.9%)	7 (11.9%)
Smoking (*n*)	Current smoker	3 (2.8%)	2 (4.1%)	1 (1.7%)	1 (2%)	2 (3.4%)
	Never smoker	58 (53.7%)	22 (44.9%)	36 (61%)	29 (59.2%)	29 (49.2%)
	Former smoker	47 (43.5%)	25 (51%)	22 (37.3%)	19 (38.8%)	28 (47.5%)
Tobacco Use (packyears)		18.0 (5.0:33.5)	25.0 (15.0:43.8)	10.0 (3.2:22.2)	10.0 (3.2:15.0)	25.0 (9.8:43.8)

Abbreviations: BMI: Body Mass Index, CRP: C-Reactive Protein, DL_CO_: diffusing capacity of the lungs for carbon monoxide, IQR: interquartile range, *n*: number, suPAR: soluble urokinase Plasminogen Activator Receptor. Packyears of tobacco use only include current or former use of tobacco. CRP and leukocyte cell count values were available for 72 patients, and suPAR estimates were based on available measures from 66 patients.

**Table 2:** Pulmonary function variables at the respiratory outpatient clinic 4–5 months post-discharge.

Respiratory Outpatient Clinic		
Variable		Mean (SD)
FEV_1_ (%)		90.4 (19.7)
FVC (%)		94.3 (17.6)
DL_CO_ (%)		78.9 (20.5)
TLC (%)		87.6 (14.5)
		Number (% of total)
FEV_1_ *n* (%) (*n* = 109)	<80% predicted	26 (23.9%)
	>80% predicted	83 (76.1%)
FVC, *n* (%) (*n* = 109)	<80% predicted	17 (15.6%)
	>80% predicted	92 (84.4%)
FEV_1_/FVC, *n* (%) (*n* = 109)	<0.70	15 (13.8)
	≥0.70	94 (86.2)
TLC, *n* (%) (*n* = 110)	<80% predicted	31 (28.2%)
	>80% predicted	79 (71.8%)
DL_CO_, *n* (%) (*n* = 110)	<80% predicted	50 (45.5%)
	>80% predicted	60 (54.5%)

Abbreviations: DL_CO_: diffusing capacity of the lungs for carbon monoxide; FEV_1_: forced expired volume in the first second; FVC: forced vital capacity; SD: standard deviation; TLC: total lung capacity. One missing measure for FEV_1_ and FVC.

**Table 3:** Assessment of sensitivity, specificity, PPV, and NPV levels for DL_CO_ impairment using baseline CRP, leukocyte cell count, and suPAR cut-off values.

Variable (Units)	Cut-Off	Sensitivity	Specificity	PPV	NPV
CRP (mg/L), baseline	≥50 (low) (50–100, >100) vs. (<50)	0.52 (0.37:0.66)	0.45 (0.32:0.58)	0.44 (0.31:0.58)	0.53 (0.38:0.67)
	>100 (high) (>100) vs. (<50, 50–100)	0.22 (0.12:0.36)	0.80 (0.68:0.89)	0.48 (0.27:0.69)	0.55 (0.44:0.66)
Leukocyte cell counts, baseline (×10^9^/L)	≥3.5 (low)	0.88 (0.76:0.95)	0.07 (0.02:0.16)	0.44 (0.34: 0.54)	0.40 (0.12:0.74)
	>8.8 (high)	0.18 (0.09:0.31)	0.77 (0.64:0.87)	0.39 (0.20: 0.61)	0.53 (0.42:0.64)
suPAR (ng/mL), baseline	≥4 (low)	0.84 (0.67:0.95)	0.38 (0.22:0.56)	0.56 (0.41:0.71)	0.72 (0.47:0.90)
	>6 (high)	0.31 (0.16:0.50)	0.76 (0.59:0.89)	0.56 (0.31:0.78)	0.54 (0.39:0.69)
Combination (threshold probability)		0.97 (0.84:1.00)	0.41 (0.25:0.59)	0.61 (0.46:0.74)	0.93 (0.68:1.00)

Abbreviations: CRP: C-Reactive Protein; DL_CO_: diffusing capacity of the lungs for carbon monoxide, PPV: positive predictive value, NPV: negative predictive value, suPAR: soluble urokinase Plasminogen Activator Receptor.

**Table 4:** Distribution of CRP, leukocyte cell counts, and suPAR intervals for DL_CO_ < or > 80%.

Variable (Units)	Level	DL_CO_ < 80%	DL_CO_ ≥ 80%	Total (%)
CRP (mg/L)	<50 (low)	24 (48)	27 (45)	51 (46.4)
	50–100 (middle)	15 (30)	21 (35)	36 (32.7)
	>100 (high)	11 (22)	12 (20)	23 (20.9)
Leukocytes (×10^9^/L)	<3.5 (low)	6 (12)	4 (6.7)	10 (9.1)
	3.5–8.8 (middle)	35 (70)	42 (70)	77 (70)
	>8.8 (high)	9 (18)	14 (23.3)	23 (20.9)
suPAR (ng/mL)	<4 (low)	5 (15.6)	13 (38.2)	18 (27.3)
	4–6 (middle)	17 (53.1)	13 (38.2)	30 (45.5)
	>6 (high)	10 (31.2)	8 (23.5)	18 (27.3)

Abbreviations: CRP: C-Reactive Protein, DL_CO_: diffusing capacity of the lungs for carbon monoxide, suPAR: soluble urokinase Plasminogen Activator Receptor.

## Data Availability

The datasets used and/or analyzed during the current study are available from the corresponding author upon reasonable request.

## Supplementary Materials

The following supporting information can be downloaded at: https://www.mdpi.com/article/10.3390/jcm14051717/s1, Table S1: Assessment of sensitivity, specificity, PPV, NPV levels for DLCO impairment using baseline CRP, leukocyte cell count, and suPAR cut-off values for sensitivity analysis.
