# Supplementary material for: The Predictive Role of C-Reactive Protein, Leukocyte Cell Count, and Soluble Urokinase Plasminogen Activator Receptor for Pulmonary Sequelae in Hospitalized COVID-19 Survivors: A Prospective Single-Center Cohort Study
Source: J Clin Med. 2025 Mar 4;14(5):1717. doi: 10.3390/jcm14051717 (PMC11900503; doi:10.3390/jcm14051717)
Supplement: Supplementary file 1 [file jcm-14-01717-s001.zip › jcm-3475068-supplementary.pdf]

Table S1. Assessment of sensitivity, specificity, PPV, NPV levels for DLCO impairment using baseline CRP, leukocyte cell count, and suPAR cut-off values for sensitivity analysis.

Variable (units)	Cut-off	Sensitivity	Specificity	PPV	NPV
CRP (mg/L), baseline	≥50 (low) (50-100, >100) vs (<50)	0.51 (0.35 : 0.67)	0.45 (0.32 : 0.60)	0.42 (0.28 : 0.57)	0.55 (0.39 : 0.70)
	>100 (high) (>100) vs (<50, 50-100)	0.20 (0.09 : 0.35)	0.81 (0.68 : 0.91)	0.44 (0.22 : 0.69)	0.57 (0.45 : 0.68)
Leukocyte cell counts, baseline (x10^9/L)	≥3.5 (low)	0.90 (0.77 : 0.97)	0.06 (0.01 : 0.16)	0.43 (0.32 : 0.54)	0.43 (0.10 : 0.82)
	>8.8 (high)	0.17 (0.07 : 0.32)	0.75 (0.62 : 0.86)	0.35 (0.15 : 0.59)	0.54 (0.42 : 0.66)
suPAR (ng/mL), baseline	≥ 4 (low)	0.92 (0.74 : 0.99)	0.39 (0.22 : 0.58)	0.55 (0.39 : 0.70)	0.86 (0.57 : 0.98)
	>6 (high)	0.32 (0.15 : 0.54)	0.77 (0.59 : 0.90)	0.53 (0.27 : 0.79)	0.59 (0.42 : 0.74)
Combination (threshold probability) No pre-existing conditions[A]		0.68 (0.46 : 0.85)	0.68 (0.49 : 0.83)	0.63 (0.42 : 0.81)	0.72 (0.53 : 0.87)
Combination (threshold probability) All[B]		0.96 (0.80 : 1.00)	0.41 (0.24 : 0.61)	0.59 (0.42 : 0.74)	0.92 (0.64 : 1.00)

Calculations based on data for patients with no pre-existing Asthma or COPD.

A: threshold cut-off value based on model using only data for patients with no pre-existing Asthma or COPD.

B: threshold cut-off value based on model using data for all patients.

Abbreviations: COPD: Chronic Obstructive Pulmonary Disease, CRP: C-reactive protein, DL_{CO}: Diffusing capacity of the lungs for carbon monoxide, NPV= Negative Predictive Value, PPV: Positive Predictive Value, suPAR: soluble urokinase Plasminogen Activator Receptor.
